# Enabling change in public health services: Insights from the implementation of nurse mentoring interventions to improve quality of obstetric and newborn care in two North Indian states

**DOI:** 10.12688/gatesopenres.13134.3

**Published:** 2021-04-29

**Authors:** Sumit Kane, Prarthna Dayal, Tanmay Mahapatra, Sanjiv Kumar, Shikha Bhasin, Aboli Gore, Aritra Das, Sandeep Reddy, Ajay Mahal, Suneeta Krishnan, Michelle Kermode

**Affiliations:** 1 Nossal Institute for Global Health, Melbourne School of Population and Global Health, University of Melbourne, Melblourne, Victoria, Australia; 2 Bihar Technical Support Unit, CARE India, Patna, Bihar, India; 3 Uttar Pradesh Technical Support Unit, India Health Action Trust, Lucknow, India; 4 Population Council India, New Delhi, India; 5 School of Medicine, Deakin University, Melbourne, Australia; 6 Bill & Melinda Gates Foundation, New Delhi, India

**Keywords:** Organisational change, Implementing change interventions, Low-and middle-income country public health services, Obstetric services

## Abstract

**Background:** Few studies have explicitly examined the implementation of change interventions in low- and middle-income country (LMIC) public health services. We contribute to implementation science by analyzing the implementation of an organizational change intervention in a large, hierarchical and bureaucratic public service in a LMIC health system.

**Methods:** Using qualitative methods, we critically interrogate the implementation of an intervention to improve quality of obstetric and newborn services across 692 facilities in Uttar Pradesh and Bihar states of India to reveal how to go about making change happen in LMIC public health services.

**Results:** We found that focusing the interventions on a discreet part of the health service (labour rooms) ensured minimal disruption of the status quo and created room for initiating change. Establishing and maintaining respectful, trusting relationships is critical, and it takes time and much effort to cultivate such relationships. Investing in doing so allows one to create a safe space for change; it helps thaw entrenched practices, behaviours and attitudes, thereby creating opportunities for change. Those at the frontline of change processes need to be enabled and supported to: lead by example, model and embody desirable behaviours, be empathetic and humble, and make the change process a positive and meaningful experience for all involved. They need discretionary space to tailor activities to local contexts and need support from higher levels of the organisation to exercise discretion.

**Conclusions:** We conclude that making change happen in LMIC public health services, is possible, and is best approached as a flexible, incremental, localised, learning process. Smaller change interventions targeting discreet parts of the public health services, if appropriately contextualised, can set the stage for incremental system wide changes and improvements to be initiated. To succeed, change initiatives need to cultivate and foster support across all levels of the organisation.

## Introduction

*“After nine months what is going to happen here? Nothing. Nothing will happen, you just wait and see.” [Study participant]*

The skepticism expressed in this quote exemplifies the challenges faced by those trying to implement change interventions in low-and middle-income countries (LMIC) public health services. ‘Change’ and ‘making change happen’ have been the subject of extensive inquiries in a variety of disciplines, with scholarship from management sciences, public policy studies, and administration studies leading the way ( Beer *et al.*, 1990; Buchanan *et al.*, 2005; Pettigrew *et al.*, 2001). The consensus is that making change happen within any large organisation is difficult ( Beer & Nohria, 2000). Two broad viewpoints on change processes within healthcare settings are recognisable in the literature ( Barry *et al.*, 2018; Beer *et al.*, 1990). There are those who think that systemic change targeting organisational cultures, and not mere tinkering around the edges, is the optimal approach. Then there are those who believe that changing defined tasks and discreet processes at the frontline of health care delivery, and incrementally influencing and improving the broader system, is the most effective way to achieve enduring change. Irrespective of the perspective, there is agreement that making change happen within large bureaucratic organisations is difficult and that change in such context occurs slowly, and only when interventions are able to shift organisational cultures. There is also a large body of research calling for more attention to the ‘human dimension’ of implementing change interventions and the need to understand the multiple layers of cultural and contextual complexity inherent in making changes in complex health systems ( Barry *et al.*, 2018; Gilson *et al.*, 2011; Montini *et al.*, 2015). Few studies have explicitly examined the implementation of change interventions in LMIC public health services; with fewer still examining how change happens in practice in such contexts, and how organizational cultures mediate implementation ( Barry *et al.*, 2018; Gollop *et al.*, 2004; Mbau & Gilson, 2018). In this paper, we seek to fill this gap by critically interrogating the implementation of two nurse mentoring interventions that sought to change and improve the quality of obstetric and newborn care services in two large North Indian states, Uttar Pradesh (UP) and Bihar. We do so to reveal *how* the nurse mentors, the central protagonists of the change intervention, along with other stakeholders, successfully facilitated and enabled improvements in labour rooms. We show how they were enabled to shift the prevailing organizational culture to bring about change. In doing so we seek to contribute to the knowledge base on implementation of change initiatives in LMIC public health services.

### Improving care in public health services: the case of maternal and newborn care in India

While significant gains have been made over the last two decades, India continues to lag many LMIC peers ( NITI, 2018) in maternal and newborn health. The main causes of maternal and newborn deaths are potentially manageable through well-functioning obstetric services ( Garces *et al.*, 2017; Say *et al.*, 2014). In India, poor quality obstetric care in public health facilities and the difficulties in changing health worker behaviour have been widely recognised ( Chaturvedi *et al.*, 2015; Das *et al.*, 2012; Das *et al.*, 2015; Sharma *et al.*, 2017). Evidence suggests that inadequate financial and human resources, and poor health system management and governance intersect with broader socio-politico-cultural environments to create conditions whereby changing behaviours becomes particularly difficult to achieve – regularly evoking reactions laden with frustration, as expressed in the quote at the beginning of this paper. Specific to the provision of midwifery services, a few issues stand out. In India, midwifery training for nurses posted to labour rooms is often inadequate – nurses do not gain the basic midwifery competencies recommended by the International Confederation of Midwives ( Sharma *et al.*, 2015). Inadequate on-the-job training, lack of supportive supervision, poor supplies of equipment and drugs, and high workloads further contribute to poor performance ( Sharma *et al.*, 2017).

To improve the quality of midwifery services, several Indian states have implemented programs that involve mentoring of labour room nurses in public health facilities ( Das *et al.*, 2016; Raney *et al.*, 2019; Semrau *et al.*, 2017; Singh *et al.*, 2016; Varghese *et al.*, 2019). Common features of these programs include on-the-job peer-to-peer mentoring and the systematic use of simple tools (e.g. checklists or case sheets) for every delivery. A recent large randomised controlled trial examined the impact of a checklist-based nurse coaching program on maternal morbidity and mortality; while this relatively short-term intervention could not show impact on these outcomes, it did significantly improve the uptake of evidence-based practices ( Semrau *et al.*, 2017). Evaluations of other nurse mentoring programs have similarly achieved improved uptake of evidence-based practices by labour room nurses ( Das *et al.*, 2016; Das *et al.*, 2017; IHAT, 2017; Washington *et al.*, 2016).

In this paper we critically interrogate the implementation of two nurse mentoring programs in Uttar Pradesh and Bihar, to examine how the nurse mentors, with active support from a range of stakeholders, managed to navigate the many challenges encountered during implementation of the programs. We contribute to the field of implementation science on two fronts. Firstly, we analyse and describe *how* these nurse mentors, who initially encountered overt and covert resistance, managed to successfully facilitate change in labour room practices and behaviours. Such implementation insights are relevant to the design and scale up of future nurse mentoring initiatives in India, and in similar resource-constrained settings. Secondly, we infer lessons to add to the knowledge base on implementing change interventions in large organizations like public health services, in India and other similar LMIC health systems.

## The nurse mentoring programs in Uttar Pradesh and Bihar

The two nurse mentoring programs (hereafter referred to as the programs) were implemented through dedicated Technical Support Units (TSU) in close partnership with the respective State Governments and were one among several initiatives implemented by the TSUs to support State Government efforts to improve reproductive, maternal, newborn and child health. Evaluations of both programs have revealed improvements in the uptake of several evidence-based practices by nurses. In UP, the Rolling Facility Survey tracked the performance of the program across 45 health facilities across 100 blocks (sub-districts) of 25 districts, across five good labour room practice domains, using a wide range of indicators; this included direct observation of 535 deliveries in Round 1 and 604 deliveries in Round 2. While the level of improvement varied across domains, there were improvements in the uptake of evidence based practices across the board; for example the survey found that active management of third stage labour score improved by 43 percentage points and essential newborn care practices by 46 percentage points between the first round in 2015 and the second round in 2016 ( IHAT, 2017). Detailed evaluations of the nurse mentoring program in Bihar (see: Ghosh *et al.*, 2019; Rao *et al.*, 2019) also show consistent improvements in labour room practices. For instance, the mentored nurses performed 17.5% more correct actions for normal delivery, 25.9% more correct actions for postpartum hemorrhage, and 28.4% more for neonatal resuscitation than the comparison group, with the mentoring effect being statistically significant for all. While it is beyond the scope of this paper to delve into a detailed presentation and analysis of process, output, and outcome data for the two states – this analysis can be accessed elsewhere, and directly through the authors. To reiterate, the focus of this paper is limited to exploring and understanding how the nurse mentors navigated the many challenges encountered during implementation of the programs, and how they overcame the challenges to successfully effect change in behaviours and practices in the labour rooms within public services.

### Program (intervention) locations – study sites

UP and Bihar together are home to more than 300 million people. Of the 29 States and 7 Union Territories of India, they consistently report among the lowest human development index and among the highest maternal mortality in the country ( GDL, 2018; NITI, 2018; Registrar General of India, 2017). Socio-culturally the two states are characterised by a feudal agrarian economy, and a deeply patriarchal society divided along entrenched caste-class-ethnic lines ( Rasul & Sharma, 2014). At the time of program inception, a range of additional problems constrained the implementation of quality improvement interventions in both state health systems: supply of drugs and equipment was irregular, infection control practices were inadequate, and human resource shortages were commonplace with labour rooms often staffed by only one or two nurses per shift, with limited support from doctors in the event of obstetric emergencies ( NITI, 2018; Vail *et al.*, 2018). In addition, the organisational culture tended to foster poor communication between staff, hierarchical relationships (with nurses near the bottom of the hierarchy), and unsupportive management approaches ( Varghese *et al.*, 2018). To say the least, the two states represented very challenging contexts to implement any change initiative.

The program in UP was implemented in 25 districts across 350 primary and community health centres. In Bihar, the program, known as *Apatkaleen Matritva Evam Navjat Tatparta* (AMANAT), intervened in 320 facilities providing Basic Emergency Obstetric and Neonatal Care (BEmONC) and 22 facilities providing Comprehensive Emergency Obstetric and Neonatal Care (CEmONC). The analysis presented in this paper relates to the implementation of the intervention at public facilities providing BEmONC services in the two states.

### Program design

In both states, the program was embedded within state public health services - led by public officials of the TSU. The intervention involved ongoing bedside supportive supervision and regular skill building sessions of labour room nurses to improve knowledge, skills and practices related to labour, delivery, and essential newborn and post-natal care, and promotion of a safe delivery checklist for every birth. Specifically, the intensive training included: refreshers on practical skills in maternal and newborn health care; placement in a labour room for a week to conduct deliveries in the contexts they would work in; communication skills; use of decision-support tools such as checklists; sessions on organizational culture, quality improvement, and change management. In both programs the mentors were qualified nurses, were women, were usually younger than the average mentee (and therefore sometimes with less professional experience). In both states the nurse mentors were hired from outside the public system and were trained and deployed exclusively for the program. Both programs provided training to prepare mentors for their roles, and as elaborated further later, mentors received ongoing support (e.g. guidance on technical matters, assistance with problem solving and dealing with bureaucratic processes) from the TSU teams. In UP the approach involved periodic meetings with the mentors to support and assist them to solve their problems, and on annual refresher trainings for new guidelines or other topics selected by the mentors. In Bihar, the approach was slightly different as each nurse mentor had a ‘master mentor’ she could turn to for support and advice as required. Finally, the TSUs in both programs took concurrent actions to improve availability of drugs, supplies, and equipment.

The differences in the design and implementation of the two programs reflected the unique circumstances in each state. For example, the mentors in UP were recruited locally whereas the mentors in Bihar hailed primarily from outside the state. This was necessary because very few graduate nurses were available in Bihar when the mentoring program was initiated. Mentors in UP had an ongoing role and were placed in and based at public health facilities. Whereas in Bihar, they provided mentoring through monthly one-week long visits over an eight-month period. *Extended Data* File 1 ( Kane *et al.*, 2020) provides details of the two programs.

## Methods

### Study design and framework

Data are drawn from a broader inquiry that compared implementation processes across nurse mentoring programs in UP and Bihar. This inquiry was guided by the Comprehensive Framework for Implementation Research (CFIR) ( Damschroder *et al.*, 2009) and included a wide-ranging examination of what worked, where, how and for whom, when implementing the nurse mentoring program in different contexts. The CFIR identifies five domains (intervention, inner setting, outer setting, processes, and individuals) that interact in complex and dynamic ways to collectively influence the effectiveness of program implementation and outcomes. *Extended Data* File 2 ( Kane *et al.*, 2020) presents the framework for the broader inquiry and an overview of how the framework was used; it also presents a synthesis of key findings emerging from the broader inquiry. In this paper we focus our analysis on two TSU-based nurse mentoring programs from the broader inquiry. We interrogate the data with the aim of providing a comprehensive, integrated understanding of how the nurse mentors overcame a wide range of barriers to successfully effect change in behaviours and practices of labour room nurses. Our intention is to spotlight the mentors’ exercise of agency when overcoming the challenges they encountered. Our analysis of the mentors’ experiences and approaches helps reveal how change occurred in practice. We signpost implications for future organisational level change interventions in similar settings. 

### Study sites

We purposively selected high- and low-performing public health facilities; performance of facilities was gauged by TSU staff based on their own program metrics recorded during routine TSU program monitoring and evaluation at the time of the study; these metrics were related to uptake of specified evidence-based practices by labour room nurses, such as administration of oxytocin, and skin-to-skin contact as per protocol. The study was conducted in eight facilities across seven districts in UP (four high performing and four low performing), and ten facilities across nine districts in Bihar (four high performing, four low performing and two facilities that had not been classified at the time of data collection). Table 1 gives an overview of the facilities. While the sampling of study sites was organized along these lines, the purpose was not to compare high and low performing facilities but rather to better understand the differences if any, across sites with differing program performance and outcomes. While we did find a range of factors that explained the difference between facilities characterised as high versus low performing, and these have been summarized in Extended Data File 2, for the specific line of inquiry presented in this paper, it emerged that there were no real differences in the explanations of how the nurse mentors navigated change and overcame the barriers that they faced. In fact, a consistent picture emerged across all facilities across the two states. 

**Table 1.** Summary of public health facilities visited for data collection.

District	Facility type
**UTTAR PRADESH**	
Faizabad	CHC
Barabanki	CHC
Farrukhabad	CHC x2
Pilibhit	CHC
Kannauj	CHC
Kheri	CHC
Kasganj	CHC
**BIHAR**	
Banka	CHC
Sitamarhi	CHC
Rohtas	CHC
West Champaran	CHC
Sheikhpura	CHC
Vaishali	CHC/PHC
Aurangabad	CHC
Patna	SDH
Saran	PHC

CHC: Community Health Centre; PHC: Primary Health Centre; SDH: Sub-district Hospital.

### Study participants

We conducted in-depth interviews with all labour room nurses (staff nurses and ANMs) who had been exposed to the nurse mentor program and who were present at the facility at the time of our visit. In UP, all nurse mentors linked to the selected facilities were interviewed. In Bihar, because the mentoring program had finished 6 months prior to data collection, a smaller number of mentors were available for interviews. We also interviewed doctors and other staff linked to the nurse mentoring program at the selected health facilities. Additionally, members of the TSU management teams and monitoring & evaluation teams were interviewed. Some interviewees identified other potential informants as sources of valuable insight – some of them were subsequently interviewed. Table 2 provides an overview of study participants and number of interviews conducted.

**Table 2.** Summary of interview participants.

Category of participant	# Uttar Pradesh	# Bihar	Total
TSU leadership and management	7	7	14
Nurse mentors	8	4	12
Monitoring & evaluation team members	1	3	4
Labour room nurses	20	35	55
Doctors *	10	6	16
Others **	4	9	13
Total	50	64	114

* Doctors include Medical Officers In-Charge and Lady Medical Officers

** Others include pharmacists in UP and sub-district managers, block managers and block health managers in Bihar.

The study sample size was determined by the availability of medical staff, mentors, and eligible mentees at each facility on the days we visited. Data collection often involved the researchers travelling long distances to remote areas of different districts (sometimes involving overnight stays); the amount of time that could be spent in each district and at each health facility, thus varied.

### Recruitment

Medical Officers In-Charge (MOICs) of health facilities were informed about the study. TSU leadership notified MOICs that study team members would be visiting the facility on a given date to interview staff (nurses and doctors). Given the fact that the TSUs were integrated into the state health services and the MOICs were very familiar with their activities, the study team was able to readily obtain access to study sites and participants.

### Data collection

The in-depth interview guides were initially drafted in English and subsequently translated into Hindi, back-translated, and piloted in the field. The mentee interviews were mostly with individual nurses, but on a few occasions some of the nurses asked to be interviewed together, which meant 2-3 nurses were interviewed at the same time. All interviews were conducted in a private location, with only the interviewers and participants in attendance. The voluntary nature of participation was carefully explained, but no participant declined to participate. Interviews were conducted by four researchers (authors PD, PR, SB, and MK) working in pairs. Interviews were conducted in Hindi or English according to the comfort of the participant; of the four data collectors, three were fluent in Hindi (the local language). All the mentees were interviewed in Hindi, and most of the medical and TSU staff were interviewed in English, as were some of the Bihar mentors who originated from South India. Each interview lasted between 30 minutes to one hour. All interviews were digitally audio recorded, and subsequently translated and transcribed verbatim into English by bi-lingual Hindi-English speakers with previous transcription and translation experience. All transcripts and translations were independently cross-checked against the original audio recordings for accuracy by authors PD, MK, and SB.

### Data analysis

We conducted an iterative thematic analysis of the data ( Braun & Clarke 2006). Following discussions at the end of each interview and the end of each day of fieldwork, review of audio files, and multiple readings of verbatim transcripts, we arrived at a set of preliminary codes. Verbatim transcripts were then coded using OpenCode 4.03 and Atlas Ti [version 6.2]. We held regular discussions to refine and specify the codes and to arrive at themes. Further, during the analysis and while writing the paper we revisited the data and iteratively developed the arguments presented in the findings and discussion sections. This paper primarily draws on codes related to ‘how’ the intervention was implemented by the nurse mentors, and how they functioned and facilitated improvements in labour rooms.

### Ethical approval

Ethics approval for this study was obtained from the University of Melbourne Human Ethics Sub-Committee in Australia (Ethics ID 1749449), and the ethics committee at the Post-graduate Institute for Medical Education and Research in Chandigarh, India. All prospective participants were provided with an information sheet that explained the study’s scope and purpose, and all participants gave informed written consent to participate and allow recording of the interviews. Audio files and verbatim transcripts were anonymised.

## Results

We present our findings as thematic accounts of how the mentors navigated and drove the change processes. In proffering explanations of mentors’ actions, we refer and link back to the hierarchical, resource-constrained health system, and the socio-cultural contexts the interventions were embedded within. This is followed by an account of the program design and resources that enabled the mentors to effect change. As indicated earlier and as elaborated in the Extended Data File 2, while there were differences between high and low performing facilities e.g. staffing levels, staff transfers etc, the explanations for how the nurse mentors coped and overcame barriers were similar across settings.

### Navigating the system, driving change

***Overcoming their ‘outsider’ status***. The nurse mentors were perceived as ‘outsiders’ by many study participants, including the mentors themselves, because they were not formally part of the government health services, and in the case of Bihar, also hailed from different parts of India. The perception of the mentors as outsiders combined with the fact that they were often younger than the mentees was regularly invoked by all participants as an explanation for the mentors’ initial struggle to be accepted by their would-be mentees, and by the system. All interviewed mentees (35 in Bihar, 20 in UP) gave positive accounts of their relationship with the mentors – they were unanimous in highlighting that they respected and valued their mentors. This respect for and appreciation of the mentors was echoed by the doctors and the MOICs at the facilities. However, as the following excerpt illustrates, this acceptance was hard earned.

*The Nurse Mentors who came before us had to be extremely patient because it was even difficult to talk to them* [the mentees] *. They* [the mentees] *scolded us, but we never backed out. They did not allow us to enter the labour room and they would not even provide us with a chair because we were not government employees. But now the rapport is so good that they are giving us chairs, rooms and … they even share their lunch with us. (Nurse Mentor, UP)*

For the mentors, the process of overcoming their outsider status was deliberate and ongoing. They recognised that being included and accepted was going to be a hard-earned privilege. The careful cultivation and maintenance of this privilege was an overarching feature of ‘how’ the nurse mentors functioned. This relational dynamic underpinned, sometimes explicitly, but usually tacitly, how they went about effecting change. This dynamic in many ways provided the scaffolding for the other approaches (described below) taken by the mentors to trigger, facilitate and support small and large changes to the status quo.

***Building rapport and earning trust by treating nurses with respect and dignity***. The accommodating and supportive approach of the nurse mentors was an important reason for their ultimate acceptance. The mentors were always polite and treated the labour room nurses with the respect that one would accord to someone who is older, and as the quotes below signal, this was an important relational expectation in the societal contexts of UP and Bihar. Similarly, the mentors were respectful of the nurse’s workload and time. During their initial training and through ongoing supportive supervision, the mentors were encouraged and supported to build constructive relationships with the nurses and facility staff. All this was a departure from the unsupportive and at times hierarchical and adversarial work culture that most nurses were accustomed to. For example, instead of invoking their authority to demand deference, the mentors conducted their activities in an accommodating and flexible manner.

*We are older. If we make any mistakes, then they explain to us very kindly that this is the right way to do this thing. Sometimes I would say, let me do my work, I’m busy. Even then she wouldn’t leave. She would say … ‘didi’* (elder sister) *first correct your mistake and learn the right way, then continue your work. I would agree. She is very nice. [Nurse, Uttar Pradesh]*

Achieving such respectful relationships was easier said than done. The mentors had to carefully navigate the existing culture and chain of command at the facility while at the same time making sure they did not condone or participate in the more punitive features of the prevailing culture. For them to operate effectively, as the following quote illustrates, the mentors had to ensure that their actions were not seen as an overt attempt to undermine the traditional chain of command.

*I started building rapport by keeping the issues limited to me and my team and then discussing it with Sir (MOIC) and them. I never did anything where someone would be scolded. [Nurse Mentor, Uttar Pradesh]*

***Recognizing and empathizing with the nurses’ workplace challenges***. Nurses in many places (including in India) work in harsh conditions, are relatively less empowered in the health system, and yet are expected to shoulder heavy workloads and deliver good outcomes for patients with minimal support. A mentor illustrated this point with the following comment “ *when we came, we saw that there were a lot of problems … there was such a huge workload … they didn’t even have electricity. There are no inverters here and so they have to work the entire hot nights by burning a coil of Mortein (mosquito repellent) to protect themselves from mosquitoes.”* As the following excerpt highlights, mentors recognised the importance of empathizing with the difficulties faced by the labour room nurses.

*Earlier they felt that everyone just came and said that the staff were not working properly. Everyone … blamed it on them. Then we came and we took their side … they felt that there was someone who understands them because our background is nursing as well. [Nurse mentor, UP]*

Mentors recognised many of the small and large routine challenges faced by labour room nurses. In their role as nurse mentors, they were empowered to go beyond expressions of empathy, to work together to take actions to address many of the problems. As the following excerpts illustrate, the mentors listened to the nurses, heard their frustrations, and advocated for them, thereby giving them a voice.

*So, I told MOIC Sir that if our staff are shouting, it means that they are in need of something ... she too needs a good environment to work. [Nurse mentor, UP]*
*Earlier no one would pay attention ... like for medicines, or if we talked about cleanliness, no one would pay attention. Even the sweeper would not listen to us. Since the Nurse Mentor has come, all this has become better. [Nurse, UP]*

This empathetic approach was a refreshing change for the nurses. Once they saw that the mentors did not mean to blame them for poor performance, they were more open to the mentoring and ultimately, to changing their practices. Mentors quickly identified that many labour room nurses felt helpless due to ongoing lack of support and recognition. Understanding the challenges faced by mentees and recognising the achievements of mentees was a key message in the mentors’ training. These messages were regularly reinforced by TSU staff during their supervisory visits.

***Modelling new behaviours***. Modelling new behaviours occurred at several levels. The nurse mentors were able to demonstrate and model safe practices while working alongside nurses in the labour room, assisting with deliveries. When asked what change if any had occurred as a result of the mentoring, a nurse in UP said that earlier they would always be worried about what to do, especially when complications such as post-partum haemorrhage arose; adding that “ *now we feel that we are strong enough and we can handle the situations.”*

The nurse mentors were also able to model assertiveness, confidence and self-efficacy. Most nurses in India are women and the broader sociocultural context of UP and Bihar is such that nurses operate within and are products of a patriarchal society that systematically discourages women’s exercise of agency and overt demonstrations of self-efficacy ( Kasturi, 1996; Kakar & Kakar, 2007). The nurses’ training reinforces deferential attitudes towards hierarchical authority (especially doctors). This was a key hurdle for the nurse mentors to overcome. The nurse mentors created safe spaces that encouraged nurses to embody confidence and enact self-efficacy. They were able to do this not only by imparting knowledge and skills, but also by being a positive role model. Although always respecting the authority of the doctors and other staff, the nurse mentors were neither obsequiously deferential, nor afraid to speak up. As the following quote illustrates, mentors were able to convincingly demonstrate to the nurses the possibility and benefits of working together as a team and supporting each other.

*First (foremost) … they taught us how we could solve our problems. Earlier we were always worried … we were very scared whenever a serious case came through. But with this, what happened is that whatever case comes, we are not scared, and we (should) all collectively work on it. We learnt about how to manage … and how to seek help ... We understood all this. [Nurse, Bihar]*

Leading by example, the mentors promoted the value of taking responsibility for one’s own work. This was in sharp contrast to the situation where the norm was for nurses to do the minimum necessary, and when things were unavailable, or when they were unable to handle a particularly complicated clinical situation, to focus primarily on sheltering themselves from blame. A nurse in Bihar summarised the prevalent attitude *as “before … it would be like … I have duty from 8 to 2 ... my duty will finish. When the other nurse would come at 2, we would just tell them, there's a patient look at her, I am leaving … That is what we'd do, once our duty was over.”* She contrasted this with the change that had occurred since their interactions with the nurse mentors, pointing out that now the nurses *“take responsibility... We give the handover, we fill in the handover book that these are the medicines, this is over, this we have used, this is left to give... and the patient is in this state, the dilatation is this much. We tell everything and go; we do handover with full responsibility”.*

The nurse mentors influenced change not only in labour room practices, but also more broadly. Mentors were able to inculcate a sense of pride in work and ownership of the workplace. Many nurse mentors across both programs reflected on how they themselves swept and washed the labour rooms to show nurses how pride in one’s workplace can generate a sense of purpose and reward. In the following excerpt a nurse reflects on how her approach to work and to her workplace changed.

*We have learned about the dignity of labour from them …. if we can sweep our home, why can’t we do that in labour room. We ourselves clean the labour room. Earlier we had the attitude like … why should we do all these things. Now we keep the labour room clean and arranged just like our homes. Earlier we only used to do duty but now … we also take responsibility for everything. We don’t have stress in doing all these things, we do it happily [Nurse, Bihar]*

The mentors modelled professionalism, commitment to delivery of good quality care, constructive assertiveness, and self-efficacy. Leading by example, as illustrated in the excerpt above, was complimented by creation of opportunities for nurses to imbibe and enact the modelled practices and behaviours, which was a key driver of change and improvements in the quality of care.

***The value of being present***. The mentors’ reliable presence empowered and enabled the labour room nurses to make changes to labour room practices. Both programs were designed so that the nurse mentors spent extended periods of time with their mentees in the labour room. The presence of the mentors in the labour room and in the facility generally, provided the nurses with much needed ongoing support, especially when the workload was high or when confronted with women experiencing complications.

*Because of madam* (nurse mentor) *we always feel like there is someone to support us. If you are doing any work, and there are some supporters, then your courage increases. Support gives us confidence that we will surely be able to do it, and ma’am always supports us. [Nurse, UP]*

The presence and availability of the nurse mentor facilitated the inculcation of what had been taught. The support of the nurse mentor was experienced by the nurses even when she was not physically present. The following anecdote is a compelling example of the strength of commitment on the part of some of these young nurse mentors (going well beyond the call of duty in this example). The anecdote not only highlights the approachability, trustworthiness and presence of the nurse mentor, it also signposts the limited support that nurses tend to have when faced with difficult situations.

*One night at 2 am, I received a call from one of my facilities. The nurse was on night duty and she asked me Ma’am, one pre-eclampsia case has come, and I would like to administer Magsulf (Magnesium Sulphate). It was 2 am … I was in deep sleep ... but I said, yes ‘Didi’ (elder sister) you must do it. Then she took a picture on WhatsApp to show me the ampoule, the dose, how she loaded it. Then I said, ok give it. The doctor was not taking any responsibility and said you and your mentor take responsibility. I said, yes, I am taking the responsibility. The nurse said, ok if you are encouraging, then I will go for it. She administered it, and the baby was delivered safely. [Nurse Mentor, Bihar]*

### Making learning a positive experience

Nurse mentors strived to make learning a positive experience through the deliberate creation of safe spaces for nurses to clarify doubts (repeatedly) without being judged and without fear of reprisal. This was central to building confidence amongst the mentees. The patient approach of the mentors is evident in the following quotes, this approach foregrounded much of the change that the mentors were able to achieve.

*She doesn’t get angry, she doesn’t get irritated and no matter how many times we ask her a question, she will answer it every time without being irritated*. *[Nurse, high performing facility, UP]*
*She never got angry at us, never said that I have already explained this, and will not tell now. She always willingly explained things and cleared our doubts. [Nurse, Bihar]*

Several nurses reflected on how much they enjoyed the training provided by the nurse mentors and how they had gained more interest in their work. One nurse in Bihar, reminiscing about her experience, said “ *we had a lot of fun … and we learnt as well”.* Another nurse in Bihar described the trainings and explained why they were so enjoyable.

*We played games as well, and if we made a mistake, we had to dance. If we could not answer, we were called out, and we had to either sing or dance. It was fun. After lunch, a game was played so that people don’t fall asleep and after that we went for simulation. [Nurse, Bihar]*

As the above quote illustrates, the program adopted a relaxed, participatory approach to learning, which was a departure from the more traditional teaching approaches the nurses were accustomed to. This approach together with the mentors ongoing presence and the provision of a safe environment to apply and practice new knowledge and skills, made learning a positive experience for the labour room nurses. Nurses were therefore more open to changing practices.

These findings beg the question ‘Why were these mentors (themselves nurses) able to treat their peers differently and how were they able to model new behaviours?’ One possible explanation is that as outsiders, they needed to exert efforts to establish trust in order to perform their role successfully, a role for which they were being held accountable by an agency (the TSUs) endorsed and overseen by the government. As outsiders, they were not directly beholden to the dysfunctional aspects of the internal organisational culture in the same way as the facility staff were, and therefore not so influenced by the hierarchy and power dynamics that tended to disempower the nurses and contribute to poor quality care. In the next section we further analyse how the mentors’ training, the ongoing support from the TSU, and the institutional arrangement of the program created conditions that enabled nurse mentors to exercise agency and to effect change. 

### Enabling nurse mentors to be agents of change

Given the demanding nature of the nurses’ work environment in these settings, the changes achieved by the nurse mentors would not have been possible without enabling conditions at the organisational level. The nurse mentoring programs by design, and by virtue of their adaptability and openness to the emerging ground realities, enabled the mentors in a range of ways.

***Preparing the mentors for a challenging environment***. As indicated earlier, both programs invested substantial time and effort in preparing mentors for their role as change agents. As the following quote illustrates the mentors valued the practical skills in maternal and newborn health care, the communication skills, and the sessions on organizational culture, quality improvement, and change management. Many told us that without these inputs they would not have been able to do their work effectively.

*We learned a lot (in the training). It feels good, that we know these things. […] The people (the mentees) have experience equal to our age, so how to deal with them, how to talk to them and how to explain things to them. How to point out their mistakes so they will not feel bad – all these things were taught to us very well. [Nurse mentor, UP]*

The comprehensive pre-service training prepared the mentors to enter the demanding environment of the state public health services and the facilities. But it was the ongoing training and support from the TSU actively focused on building confidence to engage effectively with the facility leadership, that contributed to the ultimate effectiveness of the mentors in facilitating change in what was clearly a very challenging environment.

***Time and discretionary space to navigate the challenging environment***. Any change to the status quo takes time. The TSU leadership recognised this and allowed dedicated time for the mentors to build rapport and establish credible relationships within the health facility. For instance, in UP, the first six months of the program were focused on supporting the mentors to develop relationships with labour room nurses and other facility level staff. Establishing these working relationships, as the following quote illustrates, was a painstaking exercise, but ultimately an essential and worthwhile time investment.

*I remember that when I came, I had to stand for 4 hours in front of the MOIC’s room but the MOIC did not have the time to talk to me. In fact, I cried […] I called up my team TSU leader and said that I was not at all feeling good about this … I felt insulted. But now the rapport is such that in the MOIC meetings, they comment that the nurse mentor has worked very hard and is doing well. [Nurse Mentor, UP]*

This was complemented by a program design that accorded mentors the discretion to tailor their mentoring approach to the needs of each facility, and the actors involved. While the mentors worked with a structured curriculum, they could adapt it according to the needs of the local context. Different facilities had different profiles of workers in the labour room - a mix of auxiliary nurses and general nurses, different age profiles and therefore differences in skills, interest and acceptance of the mentoring and training. Local staff profiles and labour room rotations determined how and when the nurses were available for training, and what moments and spaces were appropriate for mentoring.

***Consistent and ongoing support***. The mentors were closely supervised and supported by program implementation teams of the state TSUs. In UP, by district technical specialists and zonal technical specialists, and in Bihar by master mentors and block and district managers. The support was tactical, operational, and technical. TSU leadership was key to introducing and establishing the mentor role at both the facility and district levels. Operational support included ongoing help with accommodation, transport, and liaising with facility and district level managers to get buy-in and space to conduct their activities and catalyse the mentors’ change initiatives by negotiating improvements in infrastructure, equipment, supplies, and hospital management.

The support provided by the TSU teams, which included regular supervisory visits, was seen by the mentors as critical to their success. These visits served two broad purposes. Firstly, the visits were moments when the mentors could discuss their problems and solicit assistance. TSU team members supported the mentors directly by solving some problems and indirectly by advocating for solutions at higher levels of the public health system. The TSU visits helped to keep the mentors motivated during difficult times. Secondly, as the following excerpt illustrates, the visits also served a symbolic function; they nudged health facility staff, especially labour room nurses, to ensure they remained interested in and engaged with the mentoring program.

*If the District Technical Specialist could visit 4–5 times at our facility, currently they do 2–3 visits in 2 or 3 months … if they will do more visits then staff will remain more attentive. We are doing our work, but when outside people come for visits it makes a difference … because they (facility staff) have a mindset that those people will ask questions from them. [Nurse Mentor, UP]*

These visits communicated the seriousness of the TSU leadership’s intent; they also served to reinforce the legitimacy of the nurse mentoring program to facility level staff. In these hierarchical public health settings, this explicit operational support, and the implicit signaling that there was higher level management and institutional backing for the changes being instituted by the program, was critical to ensure that the program was taken seriously by facility level staff. These visits thus set the stage for the mentors to execute their roles and fulfil their responsibilities. The support and the supervisory visits kept the mentors motivated and ensured that they did not become overwhelmed by the health system challenges they so regularly encountered.

***Establishing and maintaining extra-organisational lines of accountability***. The discretionary authority of the nurse mentors, and the ongoing encouragement to exercise their autonomy was coupled with clear delineation of their responsibilities and unambiguous lines of authority and accountability. The program aimed to ensure that the mentors, the mentees, the facility staff and the TSU leadership, all had a common understanding on these matters. The mentors were accountable to the TSU teams and reported to TSU line managers. However, they also worked closely and respectfully with the facility MOICs, although not directly answerable to them. This relational arrangement emerged from a deep and nuanced understanding of the organisational culture of the public health services in these two states.

All health facility staff at the PHC and CHC levels, be they regular employees or contract staff, ultimately report to the MOIC. The MOIC has discretionary authority over all operations within the health facility, including assignment of tasks to anyone on the premises. Theoretically, an MOIC could ask a nurse mentor to do things that s/he deemed necessary for the facility – thereby removing her from the mentoring role. By clearly delineating responsibilities and establishing and delicately maintaining new extra-organisational lines of accountability, the TSU teams in UP and Bihar averted this problem and created and maintained an environment that assured ongoing cooperation from key actors within the health services.

## Discussion and conclusions

In this section we summarise what we have learned about how the nurse mentoring programs were able to effect change. These learnings are highlighted in Box 1; they are discussed in light of the extant theoretical work on making change happen in large bureaucratic organisational settings. Reflections are focused on implications for change management initiatives in LMIC public health services.

Box 1. **Key Learnings**

**Drivers of programmatic success**

Focusing on a clearly defined and contained area of health care (i.e. labour rooms) allowed the mentors to facilitate change that was manageable and meaningful, and a clear demonstration of the possibility for improving quality of care. This was achieved without causing undue disruption to the broader health system, which had the power to reject rather than embrace the change.

Supporting the creation of an enabling environment in terms of drugs, supplies, equipment, and job aides in the labour room so that the mentees have the means to deliver good quality care and providing space and teaching aides so that the mentors are able to foster a positive learning experience.

Allowing the mentors freedom to tailor the program according to local needs and context.

Providing comprehensive training and on-going support for carefully selected mentors.

**Drivers of mentors’ success**

Taking time to build rapport with mentees through respectful interactions, thereby earning trust.

Recognizing and acknowledging the challenges faced by labour room nurses in these contexts.

Being present and modelling new behaviours.

Providing a safe and enjoyable learning experience for mentees.

Leveraging extra-organisational lines of accountability to advocate for the nurses.

Firstly, some limitations of this study must be noted. Data saturation was undoubtedly achieved during interviews given the large number of mentees, however, due to the relatively smaller number of participating mentors in Bihar, it was not possible to judge the extent of data saturation among this group. Many of the health facilities participating in the nurse mentor program were located in remote parts of UP and Bihar, and this logistical fact shaped and constrained the extent of data collection possible. We recognise that these aspect of our approach (discreet, time limited visits, and the reliance on interviews) is a potentially important limitation of the study, and that the addition of participant observation over a longer period alongside in-depth interviewing would undoubtedly have strengthened the findings. Our understandings and inferences are shaped by our profiles as researchers. Specifically, researchers involved in data collection had diverse backgrounds, with three of the four being fluent Hindi speakers. All had extensive experience of researching health systems in rural India and were women, which possibly facilitated greater openness amongst interviewees, especially the labour room nurses and nurse mentors. The field team differed in their clinical qualifications (only one had experience as a nurse in a labour room setting), which may have contributed to slight variations in the lines of questioning and the subsequent information provided by the respondents. Researchers involved in the analysis and interpretation also had extensive prior experience working and researching health-related topics in UP and Bihar. Research findings were thus interpreted within the context of the researcher’s own prior experiences, especially the discussion about how the nurse mentors were influencing quality of care within a health system with weak lines of accountability, an enabling environment negatively influenced by gender and hierarchies, and path dependency. These are in some way also a limitation of this work.

The general consensus in the literature on ‘making change happen’ in large organisations is that it is difficult ( Beer & Nohria, 2000) – the experience of those implementing the nurse mentoring programs examined in this study echoes this consensus. While, as introduced earlier, two broad viewpoints on change processes within healthcare settings are recognisable in the literature ( Barry *et al.*, 2018; Beer *et al.*, 1990) recent literature ( Pettigrew *et al.*, 2001; Rafferty *et al.*, 2013) points to an emerging agreement around Beer et al. (1990) the view that for most large organisations, like the public health services in our study, the most effective approach is to start “at the periphery and moving steadily towards the corporate core” (p.114).

A typical LMIC public health service is a large bureaucracy ( Byrkjeflot, 2018, p.23) where members have well defined roles and responsibilities, explicit (and, also tacit) hierarchical ways of relating to each other, and well-established norms that govern the conduct of members and activities in ways that are not necessarily in the best interests of patients. The two nurse mentoring programs that were the focus of this study consciously and carefully took this reality into account; the interventions were acceptable because they did not entail major disruptions to the status quo and did not overtly threaten the established hierarchical ways of being, relating and interacting within the public health services. Deliberately and visibly ‘doffing one’s hat’ to the broader social and organisational norms of the public health service helped the nurse mentoring program to communicate a non-disruptive intent, which was essential to creating a secure and receptive environment for the change initiative to be introduced and subsequently accepted ( Rafferty *et al.*, 2013; Weiner, 2009). 

The approach taken by the implementors of the two Nurse Mentoring programs aligns with a widely recognised tenet within the vast literature on organisational culture and organisational change (see Alvesson, 2002; Rafferty *et al.*, 2013; Weiner, 2009). That those wanting to make changes need to have a good understanding of and take into account existing organisational cultures – that doing so enables the development and implementation of strategies which are appropriate for and feasible within the context ( Alvesson, 2002; Kanter *et al.*, 1992). The approach taken by the implementors of the two Nurse Mentoring programs was also aligned with Parker & Bradley’s (2000) view that such an orientation to initiating change is particularly relevant in the context of public organisations that are typically characterised by a “hierarchical-culture” that relies on formal rules and procedures as control mechanisms geared towards maintaining conformity to existing norms and cultures, and towards ensuring continuity and stability ( Zammuto & Krakower, 1991). The challenge, of course, lies in implementing changes without condoning or perpetuating existing problematic relational arrangements, practices and cultures while doing so - the nurse mentoring programs illustrate how this is possible only when one has a deep and ongoing understanding of the organisation and its context.

Our findings suggest that the intervention was able to win over most sceptics, and that many, including those with the most influence on the frontline of public sector healthcare service provision (those in leadership positions at Ministry of Health) saw the merits of different ways of functioning that the nurse mentoring program exemplified. Our analysis revealed how, to some extent, the intervention allowed frontline health workers, facility managers, and the block and district level program managers to, what Lewin (1951 p230) has called, “unfreeze” entrenched ways of being and relating, and thereby create room for change. It laid the ground for what Weiner (2009) considers “a shared psychological state in which organizational members feel committed to implementing an organizational change and confident in their collective abilities to do so”. We extend Lewin and Weiner’s viewpoints – our findings show how the presence of an explicit change agent and understanding that a mechanism to enable change in the system is in place (through the TSUs), can help shift the status quo and thereby facilitate improvements. However, we found that the organisational culture of the two public health services did not exactly unfreeze to let changes happen, but rather people and processes shifted and made room for changes. In this sense our findings are more in line with Alvesson’s (2002) critique that Lewin’s refrigerator metaphor is not particularly appropriate as it gives the impression that there is a “a frozen, i.e. fixed, social world, which can be heated up and then frozen again at will, through a correct intervention” (p.175). Alvesson argues for understanding organisational culture as something that is dynamic and constantly shifting under pressure from competing and evolving organisational logics and interests, including through the turnover of people. Our findings are consistent with his argument that it is better to instead understand the changes that occur within organisational cultures as “cultural drifting” ( Alvesson, 2002 p.176).

According to Beer *et al.* (1990), cultivating and maintaining trusting relationships with those directly affected by the change process, and those who have a stake in the change process, is essential to successfully effect change. In the context of the nurse mentoring program, this entailed the managers and the mentors consistently demonstrating commitment, coordination, and competence to all actors involved in the change process. For the mentors this involved modelling and embodying desirable behaviours, leading by example and with confidence and humility, being present in times of need, and making the change process a positive and meaningful experience for the mentees. For the program managers this involved deliberate and ongoing actions to ensure support for the program and for the nurse mentors among higher-level management, and subtly communicating this institutional backing to all key stakeholders.

For the program managers it meant consistently supporting the mentors, the central actors on the frontline of the change making process, by providing them with the hard and soft skills to act effectively, affording them discretionary decision space to adapt actions to local needs, discreetly working in the background to enable and routinize facility level changes, and taking care of some of the mentors’ day to day challenges especially those related to living away from home. These findings reaffirm the large body of evidence on organisational change and organisational readiness for change.

Finally, our findings raise some questions that deserve further reflection by researchers and those planning to implement change interventions in public health services. The mentors’ unique attributes, the approaches they took, and the systematic enablement by the TSU teams collectively made change possible. However, from an implementation perspective, that the nurse mentors and the TSU teams were outsiders to the public service bureaucracy (although endorsed by it), stands out as an issue that warrants further consideration. As findings show, on one hand being outsiders worked well for them because they were not constrained by existing hierarchical structures, but on the other hand acceptance of the mentors at the frontline was difficult because they were not government employees. The organisational change literature has extensively grappled with the dilemma of who is best placed to initiate and drive change – should it be someone from outside the organisation, or someone from within? While being outsiders worked for the nurse mentors in this study context, it remains to be seen if the same would work in other contexts. Whether having insiders (public health service staff) as mentors would be an equally successful option, is currently an unanswered question. Our findings suggest that the insertion of well-trained and well-supported external change agents who work in a respectful manner over an extended period is an effective way to help people realise the possibility of change. In contexts where organisations are moribund and dysfunctional, as was to an extent the case in the states of UP and Bihar, such a realization is important in itself as it signals to the often-demoralized frontline workers that change and improvements are possible.

Another key question is whether the changes instituted will be sustained, in the absence of an ongoing mentoring system, and what is required to achieve this goal? Evaluations of such programs have found that the effects of mentoring on nurse knowledge and practice decays over time ( Rao *et al.*, 2019). that ‘being present’ was an important mechanism through which mentors were able to facilitate change indicates that not being present would inevitably compromise the sustainability of any changes that the mentors were able to achieve. Other system factors such as the transfer of mentored labour room nurses to non-labour room functions will also interfere with sustainability. In the study contexts successful implementation of the change interventions was clearly contingent upon concurrent improvements in the health system to ensure the availability of adequate drugs, supplies, and equipment – all of which depended upon tacit and explicit support from the middle and senior management of the respective public health services. Literature suggests that change is sustained (and possible) only when senior management support initial adoption and communicate their ongoing commitment by consistently signaling, overtly and covertly, to middle managers and to frontline staff that the change initiative is an organisational priority, and demonstrate their commitment by creating an environment supportive of change ( Birken *et al.*, 2015; Dressler *et al.*, 2012; Richter *et al.*, 2016). The tension between mentors as outsiders and yet being endorsed by the government is complex and has implications for sustainability of change. While the endorsement clearly helped with the mentors being accepted, it is quite possible that this acceptance was merely another instance of the health staff and health system administrators merely doffing their hats to the whims of the higher-ups – another whim that would come to pass. While based on our data we cannot conclude either way, it is possible that the facility staff and health administrators were not particularly keen on disrupting the status quo and were only engaging with the initiative to the extent they deemed essential and sufficient to be not seen as obstructive by their higher-ups. Change endures only when senior management allocate human and financial resources to the initiative and align organisational functions such as incentives and performance reviews to enable change to be institutionalized ( Birken *et al.*, 2015; Dressler *et al.*, 2012; Richter *et al.*, 2016). We know from our experience of working within public health bureaucracies, including but not limited to in India, that these structural changes are difficult to make. Finally, whether well-resource intensive programs as the ones described in this paper can have the same results without all the enabling factors highlighted here, is yet unknown.

In conclusion, making change happen in large organisations like LMIC public health services, is possible, and is best approached as a flexible, incremental, localised, learning process. Smaller change interventions targeting discreet parts of the public health services, if appropriately contextualised and implemented at scale, can meaningfully set the stage for incremental system wide changes and improvements to be initiated. To succeed, change initiatives need to be appropriately resourced, and should cultivate and foster support across all levels of the organisation.

## Data availability

### Underlying data

All interview participants spoke with us on the explicit understanding that what they had to say would remain confidential, that only the immediate research team members would have access to the raw data, and that the study findings would be reported in summary form drawing on what all participants had to say. This was important in order to foster trust between the interviewees and the interviewers. We did not obtain permission from interview participants to make the raw data publicly available nor did we seek permission from the relevant ethics committees for this. However, if individual research peers particularly wanted to view transcripts, we are willing to consider sharing them on a limited case-by-case basis; please contact ni-info@unimelb.edu.au to make an enquiry.

### Extended data

Open Science Framework: Enabling change in public health services: Insights from the implementation of nurse mentoring interventions to improve quality of obstetric and newborn care in two North Indian states. https://doi.org/10.17605/OSF.IO/KP2A6 ( Kane *et al.*, 2020).

- Extended Data File 1 – Overview of interventions (DOCX).
- Extended Data File 2 – CFIR Framework and Its Application (DOCX).
- Interview Guides (Folder containing all interview guides; PDF).

### Reporting guidelines

Open Science Framework: SRQR Checklist for ‘Enabling change in public health services: Insights from the implementation of nurse mentoring interventions to improve quality of obstetric and newborn care in two North Indian states’. https://doi.org/10.17605/OSF.IO/KP2A6 ( Kane *et al.*, 2020).

Data are available under the terms of the Creative Commons Attribution 4.0 International license (CC-BY 4.0).
