# Supplementary material for: Enabling change in public health services: Insights from the implementation of nurse mentoring interventions to improve quality of obstetric and newborn care in two North Indian states
Source: Gates Open Res. 2021 Apr 29;4:61. Originally published 2020 Jun 18. [Version 3] doi: 10.12688/gatesopenres.13134.3 (PMC8126785; doi:10.12688/gatesopenres.13134.3)
Supplement: Peer review history

# Peer review: Reviewer response for version 3

**DOI:** 10.21956/gatesopenres.14499.r30628

**Published:** 2021-05-14

**Authors:** Sayaka Horiuchi

**Affiliations:** 1 Center for Birth Cohort Studies, University Of Yamanashi, Chuo, Japan

I appreciate that the authors have addressed my concerns. I have no further comments.

Is the work clearly and accurately presented and does it cite the current literature?

Yes

If applicable, is the statistical analysis and its interpretation appropriate?

Yes

Are all the source data underlying the results available to ensure full reproducibility?

No source data required

Is the study design appropriate and is the work technically sound?

Partly

Are the conclusions drawn adequately supported by the results?

Partly

Are sufficient details of methods and analysis provided to allow replication by others?

Partly

Reviewer Expertise:

NA

I confirm that I have read this submission and believe that I have an appropriate level of expertise to confirm that it is of an acceptable scientific standard.

# Peer review: Reviewer response for version 2

**DOI:** 10.21956/gatesopenres.14398.r29725

**Published:** 2020-10-21

**Authors:** Sayaka Horiuchi

**Affiliations:** 1 Center for Birth Cohort Studies, University Of Yamanashi, Chuo, Japan

I have read through the authors' response and revised manuscript.

 The authors have responded to many of my points successfully but I keep the status "Approved with reservations" **.**

 I have a few additional comments:

*"Introduction:*

*· **Reference should be made to the extensive literature on the role of organizational culture as it relates to phenomena of central interest in the current study (safe environment, front line empowerment, etc), especially to more precisely define the current gap and motivate the study.***

***Response: **Thank you. We appreciate your point, and we have taken this on board fully, but not in the introduction. Instead we refer to the extensive literature to more explicitly discuss the importance of taking into account and to tailor change interventions to organisational cultures and contexts. Hope this is agreeable and satisfactory."*

- Thank you for adding extensive literature and discussion in the discussion section. However, the current gap which needs to be filled in and the rationale of the current study is not clear in the introduction.

 

*"Methods:*

***5. Given there were no comparative analysis between the high and low performing sites, it is unclear why the sample was designed in this way.***

**

***Response:** Differences between the high and low performing facilities (as defined by quantifiable TSU indicators) were initially anticipated so this was used as a criteria for selecting data collection sites, but through the process of qualitative data analysis it became clear that findings from high and low performing facilities were substantially overlapping. While quantifiable indicators could clearly distinguish between high and low performing facilities, the responses to our qualitative questions did not."*

- If it was initially planned to perform comparative study between low and high performing facilities, and then results were combined after analysis, this should be explained.
- It is a bit surprising that there was no difference between low and high performance facilities. Are facilities performing low even though the mentoring system worked well as the high performing facilities? Or did mentoring not work well in the low performing facilities? It would be interesting to see discussion.

 

***"6. Given the focus on nurse mentor experiences, having only 4 in the sample for one site seems unlikely to generate saturation; please note the limitations.***

**

***Response: **The reviewer is correct, this is a limitation of the study, and is now mentioned as such in the discussion section."*

- How does this limitation influence the research findings? It would be a bit confusing to state "Our findings suggest that the intervention was able to win over most sceptics".

Is the work clearly and accurately presented and does it cite the current literature?

Yes

If applicable, is the statistical analysis and its interpretation appropriate?

Yes

Are all the source data underlying the results available to ensure full reproducibility?

No source data required

Is the study design appropriate and is the work technically sound?

Partly

Are the conclusions drawn adequately supported by the results?

Partly

Are sufficient details of methods and analysis provided to allow replication by others?

Partly

Reviewer Expertise:

NA

I confirm that I have read this submission and believe that I have an appropriate level of expertise to confirm that it is of an acceptable scientific standard, however I have significant reservations, as outlined above.

# Peer review: Reviewer response for version 1

**DOI:** 10.21956/gatesopenres.14321.r29009

**Published:** 2020-07-14

**Authors:** KR Bharath Kumar Reddy

**Affiliations:** 1 Shishuka Children's Specialty Hospital, Bengaluru, Karnataka, India

**Summary:**

 This study by Sumit Kane *et al.* is a qualitative analysis of mentor-mentee relationship and outcome of nurse mentoring interventions to improve the quality of obstetric and newborn care in two states of India. They have critically interrogated a total of 114 participants including TSU leadership, nurse mentors, labour room nurses and doctors. The study describes in detail the difficulties faced by mentors with an outsider status and beautifully elaborates on how they overcame this hurdle. We get to understand how older nurses who were being mentored by younger less experienced nurses managed to create a rapport and a relationship of trust and mentorship, overcoming the hierarchical system that exists in that state. Empathy and modeling new behaviours were found to be the main qualities which contributed in creating a connection between the two groups. The mentors also went an extra step to make sure they were always available to the mentees and made learning a positive experience. The nurse mentors had undergone adequate training not only in technical skills but also with communication skills, meandering the organizational system, quality improvement and change management. The nurse mentors also received consistent and ongoing support from the program implementation teams of the respective states. This contributed significantly to their success. This study concludes that although making a change in public health services in certain states may be difficult, it is possible with making small changes in discreet parts of the system. The article shows that for change to happen it requires support and co-operation from all levels of the system.

**Is the work clearly and accurately presented and does it cite the current literature?**

 The work is presented clearly and accurately. The headings and categorization of the sections are appropriate and well thought of.

 The study cites the latest literature in this field and is well presented in its literature search. However, a few references (for example, Beer *et al.*), are beyond 15 years old. It would be more appropriate to replace these with more recent references, preferably within the last 10 years if possible.

 The paper however seems to describe India as an LMIC with a uniform maternal and neonatal outcome. This is however not true. There are wide inter-state variations in maternal and neonatal mortality. This study is conducted in UP/Bihar which has a significantly different outcome than a southern state for example. The authors would need to comment on the nation-wide difference for readers to be able to understand the outcome of this report. Would the opinions be widely different if the same was conducted in Kerala? Yes, I would believe so. The paper needs to describe the factors responsible for wide variation in neonatal mortality in India and why it is important for such a program to be implemented in UP and Bihar and hence how the results they present are significant.

**Is the study design appropriate and is the work technically sound?**

 The study design is appropriate. The researchers have interviewed a significant number of mentees and mentors to draw the conclusions which they present systematically. The work done can be considered technically sound due to the elaborate description by the researchers on key points which need to be focused upon. As the program studied in this paper is already embedded into the public health services of the state, the design used by the researchers is applicable for this study at this particular location. However, whether the same can be replicated in another state of the country is doubtful.

**Are sufficient details of methods and analysis provided to allow replication by others?**

 The study describes the methods and analysis appropriately. However, there are a few deficiencies which could be addressed and described in the study. Firstly, there is no detailed description of where the nurses come from. Was there a difference in response based on which states these nurses were recruited from? This would be an interesting finding for future studies. Secondly, mentors in UP stayed in-house whereas those from Bihar visited for one-week each every month. What was the difference seen in the response from mentees between these two arrangements? Were the mentees and mentors able to connect with the same level of understanding in both these arrangements? It would seem that mentors who stayed in-house were more accessible and connected than those who visited. However, no comments have been made on this by the researcher, which would be required to plan further studies in the future.  

**If applicable, is the statistical analysis and its interpretation appropriate?**

 The interpretation made by the authors is appropriate and to the point.

**Are all the source data underlying the results available to ensure full reproducibility?**

 The authors need to furnish more data regarding the mentors. The demographic characteristics, educational qualifications, economic background and state of origin of the mentors could play a significant role in describing the outcome of the program. If this needs to be reproducible we would need to understand more elaborately the characteristics of the mentors to be able to say that the outcome is significant. There are no details on the involvement and role played by the MOIC other than granting permission. Why was there little involvement of the MOIC in this program? What was the reason for their resistance as described by one nurse? Would the mentors and mentees have the same rapport with a more active involvement of the MOIC? This needs elaboration for one to reproduce this study.

**Are the conclusions drawn adequately supported by the results?**

 The conclusions drawn are adequately supported by the results presented by the authors. Further elaboration of results as requested above need to be made to draw a significant conclusion.

Is the work clearly and accurately presented and does it cite the current literature?

Yes

If applicable, is the statistical analysis and its interpretation appropriate?

Yes

Are all the source data underlying the results available to ensure full reproducibility?

Partly

Is the study design appropriate and is the work technically sound?

Yes

Are the conclusions drawn adequately supported by the results?

Yes

Are sufficient details of methods and analysis provided to allow replication by others?

Partly

Reviewer Expertise:

Global health, Air Pollution, Pediatrics, PEdiatric Pulmonology, Allergy

I confirm that I have read this submission and believe that I have an appropriate level of expertise to confirm that it is of an acceptable scientific standard.

# Peer review: Reviewer response for version 1

**DOI:** 10.21956/gatesopenres.14321.r29008

**Published:** 2020-07-14

**Authors:** Sayaka Horiuchi

**Affiliations:** 1 Center for Birth Cohort Studies, University Of Yamanashi, Chuo, Japan

Overall, it is a very interesting study and the finding of chips to implement mentorship programs will be valuable to other LMICs which suffer from similar obstacles to improve quality of care.

**Comments on introduction:**

1. This appears to reach beyond the scope of the current study: “Secondly, we strengthen the knowledge base on strategies for implementing change interventions in public health facilities, in India and other similar LMIC health systems.”
2. Reference should be made to the extensive literature on the role of organizational culture as it relates to phenomena of central interest in the current study (safe environment, front line empowerment, etc), especially to more precisely define the current gap and motivate the study. 

**Study design:**

1. The qualitative methods are appropriate to address the research question. However, it was described in the Abstract that this is a mix of methods. Please change the description as this is a qualitative study. Or if the mixed method was applied, please include information on quantitative study that was combined with the qualitative study both in the methods and result parts.

**Methods:**

1. It was not clearly mentioned what types of interviews were conducted - for example: group interview or in-depth interview. How many people refused to participate or dropped out and why? Did anyone besides interviewers attend the interview - such as TSU staff who might have influence on interviewees?
2. What was the interviewer's relationship with participants? Have they known each other before the interview?
3. A number of interviews have been done. But can you provide your thought on whether data saturation was met to collect enough information to address your question? Please provide the rationale for the sample size is not provided, which is essential information.    
4. Please note any potential implications from the multiple languages and translation approaches.
5. Given there was no comparative analysis between the high and low performing sites, it is unclear why the sample was designed in this way.
6. Given the focus on nurse mentor experiences (‘how the mentors navigated and drove the change process’), having only 4 in the sample for one site seems unlikely to generate saturation; please note the limitations.
7. Please indicate high/low performing ID for the nurse mentor quotes, as is done with the general nurses, or provide an explanation as to why it is not included

 

**Comments on results:**

 The results section is well written and engaging to read, however there are areas needing clarification or expansion:

1. A limitations section is missing and is a major omission.
2. The results do not directly address adaptation or sustainability, which presumably was explored within the use of the CFIR framework, and would be key insights in terms of implementation of the models.

   

**Discussion and Conclusion:**

- It was not clear why three key learning points were drawn from the findings summarized in the result section. Especially, strategic location - it was mentioned in the results, however, it did not seem that it was a major finding. The result rather highlights a supporting environment for mentors such as training, supportive supervision and so on. Please elaborate this part in the results or provide more explanation in the discussion for why these three, especially the first one, were extracted from the results. The supporting environment seems critical here and discussions on this point and how the system would be applicable to other contexts would be helpful for readers as well.

Is the work clearly and accurately presented and does it cite the current literature?

Yes

If applicable, is the statistical analysis and its interpretation appropriate?

Yes

Are all the source data underlying the results available to ensure full reproducibility?

No source data required

Is the study design appropriate and is the work technically sound?

Partly

Are the conclusions drawn adequately supported by the results?

Partly

Are sufficient details of methods and analysis provided to allow replication by others?

Partly

Reviewer Expertise:

Maternal and child health, quality of care

I confirm that I have read this submission and believe that I have an appropriate level of expertise to confirm that it is of an acceptable scientific standard, however I have significant reservations, as outlined above.

# Response

**Published:** 2020-09-14

**Authors:** Sumit Kane

Dear Colleague,

Thank you for your constructive and detailed review of our article. We have diligently taken into account all your comments and used them to improve the manuscript. Below you will find a point by point account of how we have engaged with your comments; you will find these changes reflected in the revised manuscript. Hope that we have been able to address all your reservations to your satisfaction, and that the improvements are sufficient for you to consider upgrading your review to ‘Approved’.

All Authors

**Introduction:**

- **This appears to reach beyond the scope of the current study: “Secondly, we strengthen the knowledge base on strategies for implementing change interventions in public health facilities, in India and other similar LMIC health systems.”**

**Response: **We see your point and have revised this sentence to state that we infer lessons to add to the knowledge base on implementing change in large organizations.

 

- **Reference should be made to the extensive literature on the role of organizational culture as it relates to phenomena of central interest in the current study (safe environment, front line empowerment, etc), especially to more precisely define the current gap and motivate the study. **

**Response: **Thank you. We appreciate your point, and we have taken this on board fully, but not in the introduction. Instead we refer to the extensive literature to more explicitly discuss the importance of taking into account and to tailor change interventions to organisational cultures and contexts. Hope this is agreeable and satisfactory.

**Study design: **

- **The qualitative methods are appropriate to address the research question. However, it was described in the Abstract that this is a mix of methods. Please change the description as this is a qualitative study. Or if the mixed method was applied, please include information on quantitative study that was combined with the qualitative study both in the methods and result parts.**

**Response: **The reviewer is correct, and this research question and analysis has used qualitative data only. We have also revised this passage in the abstract to reflect this. The broader enquiry on the implementation of the nurse mentoring programs had drawn on mixed methods and this was erroneously such described in the abstract of this paper.

**Methods:**

**1) It was not clearly mentioned what types of interviews were conducted – for example: group interview or in-depth interview. How many people refused to participate or dropped out and why? Did anyone besides interviewers attend the interview – such as TSU staff who may have influenced the interviewees?**

**Response:** The interviews were in-depth and were mostly with individual nurses. On a few occasions some of the nurses asked to be interviewed together, which meant 2-3 nurses were interviewed at the same time. All interviews were conducted in a private location, with only the interviewers and participants in attendance. No potential participant refused to be interviewed, even though the voluntary nature of participation was carefully explained. This is now more clearly explained in the Methods sub-sections entitled “Study participants” and “Data collection”

**2) What was the interviewer's relationship with participants? Have they known each other before the interview?**

**Response: **The majority of the participants had never met the interviewers in a prior context. This included all nurses, nurse mentors and other health facility staff. Some of the program leaders had been introduced to some of the interviewers prior to the interview to help organize the study, but there was no other prior relationship between interviewers and participants.

**3) A number of interviews have been done. But can you provide your thought on whether data saturation was met to collect enough information to address your question? Please provide the rationale for the sample size, which is not provided and is essential information.**

**Response:** Data saturation was undoubtedly achieved during interviews with the large number of mentees. However, due to the relatively smaller number of mentors interviewed, we cannot reasonably judge the extent of data saturation among this group, and this is a limitation of the study. This point has been added to the revised limitations section of the discussion.

The sample size was determined by the availability of medical staff, mentors and eligible and consenting mentees at each facility on the days we visited. Data collection often involved the researchers travelling long distances to remote areas of different districts (often involving overnights stays), so the amount of time that could be spent in each district and in each facility was somewhat constrained by this fact. This is now mentioned in the “Study participants” sub-section of the Methods.

**4. Please note any potential implications for the multiple languages and translation approaches.**

**Response:** Three of the four researchers involved in data collection are fluent Hindi and English speakers. The interview guides were initially drafted in English and subsequently translated into Hindi, back-translated and piloted in the field. All of the mentees were interviewed in Hindi, and most of the medical and TSU staff were interviewed in English, as were some of the Bihar mentors who originated from South India. The Hindi audio recordings were transcribed and translated by bi-lingual Hindi-English speakers, and the English recordings by a medical transcriber. All transcriptions/translations (Hindi and English) were independently cross-checked by the researchers against the original recordings. More detail regarding these processes has been integrated into the “data collection” sub-section of the Methods.

**5. Given there were no comparative analysis between the high and low performing sites, it is unclear why the sample was designed in this way.**

**Response:** Differences between the high and low performing facilities (as defined by quantifiable TSU indicators) were initially anticipated so this was used as a criteria for selecting data collection sites, but through the process of qualitative data analysis it became clear that findings from high and low performing facilities were substantially overlapping. While quantifiable indicators could clearly distinguish between high and low performing facilities, the responses to our qualitative questions did not.

**6. Given the focus on nurse mentor experiences, having only 4 in the sample for one site seems unlikely to generate saturation; please note the limitations.**

**Response: **The reviewer is correct, this is a limitation of the study, and is now mentioned as such in the discussion section.

**7. Please indicate high/low performing ID for the nurse mentor quotes, as is done with the general nurses, or provide an explanation as to why it is not included.**

**Response: **We have taken out identifiers of low and high performing from all quotes. To be consistent and as described in our response to reviewer pt. 5 on methods, since similar findings emerged from the qualitative data across these sites, the ID is not relevant to the enquiry or findings of this paper.

**Results:**

**1. A limitations section is missing and is a major omission.**

**Response:** The influence of the researchers’ backgrounds and previous experiences is mentioned in the discussion section. Some limitations have been added to this paragraph, and the paragraph has been moved closer to the beginning of the discussion section.

**2.The results do not directly address adaptation or sustainability, which presumably was explored within the use of the CFIR framework and would be key insights in terms of implementation of the models.**

**Response: **We agree with the reviewer that the question of sustainability and adaptation is a vital one. We had touched on this in the discussion section and have further augmented this in our response in the revised manuscript to highlight that sustainability remains a question in the absence of an ongoing mentoring system, given the evidence that mentoring effects decay over time, and health system factors such as transfer of nurses. We do not yet know how sustainable these changes will be over time, and especially if the program is adapted without all the enabling factors described. While a more thorough discussion on sustainability is beyond the scope of this paper, this theme is being explored and presented in more depth in a follow up paper describing the results from the process evaluation using the CFIR framework.

**Discussion and conclusion:**

**It was not clear why three clear key learning points were drawn from the findings summarized in the results section. Especially, strategic location – it was mentioned in the results, however, it did not seem that it was a major finding. The result rather highlights a supporting environment for mentors such as training, supportive supervision and so on. Please elaborate this part in the results or provide more explanation in the discussion for why these three, especially the first one, were extracted for the results. The supporting environment seems critical here and discussions on this point and how the system would be applicable to other contexts would be helpful for readers as well.**

**Response: **The box summarizing key learnings has been revised so that it captures a more comprehensive range of learnings drawing on a number of findings. The revised text is as follows.

**KEY LEARNINGS**

**Programmatic success factors**

- Focusing on a clearly defined and contained area of health care (i.e. labour rooms) allowed the mentors to facilitate change that was manageable and meaningful, and a clear demonstration of the possibility for improving quality of care. This was achieved without causing undue disruption to the broader health system, which had the power to reject rather than embrace the change.
- Supporting the creation of an enabling environment in terms of drugs, supplies, equipment and job aides in the labour room so that the mentees have the means to deliver good quality care, and providing space and teaching aides so that the mentors are able to foster a positive learning experience.
- Allowing the mentors freedom to tailor the program according to local needs and context.
- Providing comprehensive training and on-going support for carefully selected mentors.

**Mentor success factors**

- Taking time to build rapport with mentees through respectful interactions, thereby earning trust.
- Recognising and acknowledging the challenges faced by labour room nurses in these contexts.
- Being present and modelling new behaviours.
- Providing a safe and enjoyable learning experience for mentees.
- Leveraging extra-organisational lines of accountability to advocate for the nurses.

# Response

**Published:** 2021-04-15

**Authors:** Sumit Kane

**Reviewer: Dr. Sayaka Horiuchi**

***Overall Comment:*** *I have read through the authors' response and revised manuscript. The authors have responded to many of my points successfully, but I keep the status "Approved with reservations" **.***

**Response: **Thank you. We appreciate the efforts you have taken to help us improve the manuscript. We have tried our best to engage with all your inputs to the revised draft (V2); hope this is satisfactory. Below, we outline how we have addressed each of your comments to V2 of the manuscript. Many changes and revisions are also in response to your co-reviewer’s inputs - you will notice this in the manuscript, and in the now majorly expanded Extended Data File 2, and in our elaborate response to your co-reviewer.

  

- ***Comment*** *: I have a few additional comments: Introduction: Reference should be made to the extensive literature on the role of organizational culture as it relates to phenomena of central interest in the current study (safe environment, front line empowerment, etc), especially to more precisely define the current gap and motivate the study.
** ***

**Response: **Thank you. We appreciate your point, and we have taken this on board fully, including in the introduction. We return to and refer to this extensive literature to more explicitly discuss the importance of taking into account and to tailor change interventions to organisational cultures and contexts. Hope this is agreeable and satisfactory.

- ***Comment*** *: Thank you for adding extensive literature and discussion in the discussion section. However, the current gap which needs to be filled in and the rationale of the current study is not clear in the introduction.*

**Response V2**: We have revised the introduction to signpost the elaborate on the rationale and highlight the gap that the study seeks to fill.

  

- ***Comment:*** *Methods: Given there were no comparative analysis between the high and low performing sites, it is unclear why the sample was designed in this way.*

**Response:** Differences between the high and low performing facilities (as defined by quantifiable TSU indicators) were initially anticipated so this was used as a criterion for selecting data collection sites, but through the process of qualitative data analysis it became clear that findings from high and low performing facilities were substantially overlapping. While quantifiable indicators could clearly distinguish between high and low performing facilities, the responses to our qualitative questions did not."

- ***Comment:*** *If it was initially planned to perform comparative study between low and high performing facilities, and then results were combined after analysis, this should be explained.*
*  *
- ***Comment*** *: It is a bit surprising that there was no difference between low and high performance facilities. Are facilities performing low even though the mentoring system worked well as the high performing facilities? Or did mentoring not work well in the low performing facilities? It would be interesting to see discussion.*

**Response**: We have further elaborated in the methods sub-section ‘study sites’, that while we organized the sampling of study sites according to high and low performance, the intention was not to conduct a comparative analysis between the two types of facilities, but rather to better understand the factors that explained program performance and impact.

 We did find a range of factors that explained the difference between facilities characterised as high versus low performing (summarized in Extended Data File 2), however for the line of enquiry of this paper, it emerged that there were no differences between the facilities in explaining how the nurse mentors navigated change and overcame the barriers that they faced to mentor the nursing staff, and broadly to make change happen. A consistent picture emerged across all facilities in both states.

  

- ***Comment*** *: Given the focus on nurse mentor experiences, having only 4 in the sample for one site seems unlikely to generate saturation; please note the limitations.*

**Response: **The reviewer is correct, this is a limitation of the study, and is now mentioned as such in the discussion section.

- ***Comment:*** *How does this limitation influence the research findings? It would be a bit confusing to state "Our findings suggest that the intervention was able to win over most skeptics".*

**Response**: We recognise this limitation generally. However, we point out and consistently highlight that the two study contexts (generally, and specifically the two health systems) are quite similar, and this became more apparent during the data collection. So, while the number of available nurse mentors in one study site was small, overall, we had a fairly large sample across the two states, with consistent findings emerging across the two states.  

 The inference above that our findings were able to win over most sceptics is not impacted by the smaller sample of nurse mentors in one study site either, as it was informed by the larger sample of nurses, and inputs from other health facility staff interviewed across the two states.

# Peer review: Reviewer response for version 1

**DOI:** 10.21956/gatesopenres.14321.r28950

**Published:** 2020-07-13

**Authors:** Lisa R. Hirschhorn

**Affiliations:** 1 Department of Medical Social Sciences, Northwestern University Feinberg School of Medicine, Chicago, IL, USA

The authors have done a well designed and written qualitative study to understand how mentorship was implemented in 2 MNH large initiatives in UP and Bihar to help understand the program successes which are briefly described. iThrough qualitative interviews, the study was designed to elicit how the nurse mentors navigated the challenges and “managed to successfully facilitate change”. I tried to follow the link to the important supplemental files such as the CFIR application, guides and implementation, but only found an overview of the interventions. While there was overlap in these 2 programs, there were differences and was surprised this was not explored in the results. Similarly for how the different components of the implementation were leveraged. This paper gives a good overview of the coaching strategy, but was done within the broader context of the interventions and importantly the resources and TSU support. My main concerns were about the need to address issues of sustainability or change and scalability. While of great interest, the amount and time of the resources needed for an intervention targeting only one maternal/neonatal period of risk should be noted as a challenge for replication as well. The discussion about how nurse mentoring programs effect change needs to be discussed in the context in these critical facilitating factors. The paper would also be improved as noted above by at least some discussion of any differences between the 2 similar but not identical programs ad exploring areas where the mentors were less effective based on the programmatic quantitative results.

 Introduction:

- In describing the outcomes of the programs, the data are described differently and it would be helpful to have the actual numbers (start and end) as well as what variability was seen (was the success in every site?). Which EBIs improved and which were resistant to change?
- The comment on page 4 about organizational culture tending to foster poor communication etc. should have a reference.

 

 Methods:

- Was any other support provided such as supplies, structural improvements, or funds to support needed changes etc?
- The reference describing the broader CFIR-based analysis (Kane 2020) does not take the reader to an analysis, only a table. Is there a synthesis available as important in which to interpret this paper
- Study sites: Was low and high performing at the start or end? Did these all change as I would think that degree of change would be an important factor to include? Why had 2 not yet been classified?
- I am also curious why one hospital was included as added in potential organizational diversity but with only 1, hard to determine.
- Study participants: The timing is a little unclear for UP-retrospective for Bihar, was that also true for UP? Was there any bias based on availability of the Bihar mentors for interviewing?

 

 Results:

- It is interesting that no difference was seen between high and low performing sites. I was hoping that the authors would discuss.
- One challenge in this model for improvement is when a mentor see a mistake not being corrected-what were the ethical protections in place of and when the mentor could/would intervene?
- When the article notes that the mentors were “empowered to go beyond “mere” (interesting choice of words as you note that empathy s important-consider reframing), to take action”-what was that-was it advocating or accessing resources? What changes were seen
- Was the impact on skills as well as the “assertiveness, confidence and self-efficacy” modelled by the mentors? Particularly relevant to the quote from UP and PPH. And the example on MagSulfate.
- Did the self efficacy improvement work in all settings or only those where MOIC or more senior nurses were (or became) welcoming?
- While the results are impressive, it would be important to understand what persisted after the mentorship ended, as other studies have found decrease in impact and some of the quotes discuss the “value of being present” for the mentors (page 7).
- There is an interesting tension about the potential value of mentors as “outsiders” (page 8) versus the perceptions in earlier sections. It is also important to note that while they may have been outsiders, they were still within the government-endorsed system (TSU) which I suspect may have helped with their acceptance.
- The findings are not surprising given the coaching literature and it would be of great value to understand how the programs chose, trained and mentored the mentors-how was supportive supervision noted in page 6 given earlier, how was the mentor performance assessed over time? The section on how the mentors were trained and supportive is critical and was a little surprised to see at the end of the results. As a reader, I kept looking for that and would have preferred to start with that and a bit more detail in the methods section. Details which are important for replication.
- The details on the “structured” curriculum would be important (perhaps a supplemental file) and example of how they adapted to meet challenges would provide deeper insight into the successes described.
- The time and resources required were substantial-is there any costing data to again inform sustainability and replication?

 

 Discussion:

- I was interested in the concept of “doffing ones hat” and how that persists as a sustainable strategy versus also working with coaching of the MOIC and others to create organizational change over time. Over time for example, did the nurses learn by example and strat advocating for change or did that remain in the domain of the mentors?
- The number of interviews is large, but facilities limited, so would be a bit cautions about the “able to win over most skeptics" - that should be caveated by who was interviewed. I also did not hear much from the MOOICs and higher up entrenched individuals about the “unfreezing” and more details would be of great value.
- The paragraph on sustainability is a great start, but let the reader wanting more details - the Bihar interviews were done 6 months after program end and given that the TSU focus was on the nurse mentors (page 10), did the interviews ask about sustainability and who is anyone was moving into a similar mentoring role?
- I was also looking for a bit more on the limitations of the approach and data. There is good discussion about the researchers and their experiences, the sustainability unknowns (see above).
- The final conclusions I think are valid, but incomplete and need to acknowledge the resources and time and the knowns about how to sustain both individual as well as the emerging organizational change.

Is the work clearly and accurately presented and does it cite the current literature?

Yes

If applicable, is the statistical analysis and its interpretation appropriate?

Not applicable

Are all the source data underlying the results available to ensure full reproducibility?

Partly

Is the study design appropriate and is the work technically sound?

Yes

Are the conclusions drawn adequately supported by the results?

Partly

Are sufficient details of methods and analysis provided to allow replication by others?

Partly

Reviewer Expertise:

Implementation research, global health and QI

I confirm that I have read this submission and believe that I have an appropriate level of expertise to state that I do not consider it to be of an acceptable scientific standard, for reasons outlined above.

# Response

**Published:** 2020-09-14

**Authors:** Sumit Kane

Dear Colleague,

Thank you for taking the time and effort to review our article. We are writing to indicate that we are working through your comments and in due course we will write to you to share our response and the changes we make in light of your inputs.

Regards

All authors

# Response

**Published:** 2021-04-15

**Authors:** Sumit Kane

**Reviewer: Dr. Lisa** **Hirschhorn**

- **Comment:** The authors have done a well-designed and written qualitative study to understand how mentorship was implemented in 2 MNH large initiatives in UP and Bihar to help understand the program successes which are briefly described. Through qualitative interviews, the study was designed to elicit how the nurse mentors navigated the challenges and “managed to successfully facilitate change”. I tried to follow the link to the important supplemental files such as the CFIR application, guides, and implementation, but only found an overview of the interventions. While there was overlap in these 2 programs, there were differences and was surprised this was not explored in the results. Similarly, for how the different components of the implementation were leveraged. This paper gives a good overview of the coaching strategy but was done within the broader context of the interventions and importantly the resources and TSU support. My main concerns were about the need to address issues of sustainability or change and scalability. While of great interest, the amount and time of the resources needed for an intervention targeting only one maternal/neonatal period of risk should be noted as a challenge for replication as well. The discussion about how nurse mentoring programs effect change needs to be discussed in the context in these critical facilitating factors. The paper would also be improved as noted above by at least some discussion of any differences between the 2 similar but not identical programs and exploring areas where the mentors were less effective based on the programmatic quantitative results.

***Response:*** *Once again, we thank you for the detailed and useful comments – we appreciate the time and effort you have taken to guide us. ****While all the points you raise are important and valuable, some of the points that you have made* *are beyond the specific focus of this paper. But we have tried to engage with all your inputs as best as* *feasible. Some of your points and concerns were already addressed in the first revision (V2) so do not obviously appear in the track changes in this version of the manuscript. We have: 1. Expanded the Extended File 2 substantially (this should be easily accessible via the OSF link given in the submission and included here for easy reference:* *https://doi.org/10.17605/OSF.IO/SXA25)* *. 2. Signposted the mechanism (for those interested) to get access to the two confidential full evaluation reports. 3. Signposted other publications that relate to these two programs. Below, we outline our reflections on each of your points* *, and indicate how we have addressed them. Hope to have satisfactorily engaged with your peer review.*

  

- ***Comment:*** *Introduction: In describing the outcomes of the programs, the data are described differently, and it would be helpful to have the actual numbers (start and end) as well as what variability was seen (was the success in every site?). Which EBIs improved and which were resistant to change?*

**Response**: The dates are presented in Extended File 1. We have added some further data too. We have also indicated that while it is beyond the scope of this paper to delve into a detailed presentation and analysis of process, output, and outcome data for the two states – this analysis can be accessed elsewhere (we have given sources) and added that it can also be directly accessed through the authors (we were commissioned by BMGF to do a third-party evaluation of the projects – this report can be accessed via BMGF). We have reiterated that the focus of this paper is limited to exploring and understanding how the nurse mentors navigated the many challenges encountered during implementation of the programs, and how they overcame the challenges to successfully effect change in behaviours and practices in the labour rooms.

  

- ***Comment*** *: The comment on page 4 about organizational culture tending to foster poor communication etc. should have a reference.*

**Response**: Thank you. We have added a reference which points to the state of the organizational culture and how it relates to nurses. Varghese et al 2018.

  

- ***Comment:*** *Was any other support provided such as supplies, structural improvements, or funds to support needed changes etc?*

**Response**: No additional resources were provided. But as indicated in the manuscript, the TSUs facilitated the availability of supplies etc.

  

- ***Comment*** *: The reference describing the broader CFIR-based analysis (Kane 2020) does not take the reader to an analysis, only a table. Is there a synthesis available as important in which to interpret this paper.*

**Response:** The Extended File 2 has bene expanded to now include the analysis. Again, the larger report contains further details – it cannot be publicly shared given the agreements with the program partners. Interested parties can however reach out to the listed BMGF co-authors for a not-for-circulation copy.

  

- ***Comment:*** *Study sites: Was low and high performing at the start or end? Did these all change as I would think that degree of change would be an important factor to include? Why had 2 not yet been classified?*

**Response:** We have elaborated upon this further in current manuscript. While the sampling of study sites was organized along the lines of high and low performing facilities (as assessed by one metric), the purpose was not to compare high and low performing facilities but rather to better understand the differences if any across sites with differing program performance and outcomes.

  

- ***Comment*** *: I am also curious why one hospital was included as added in potential organizational diversity but with only 1, hard to determine.*

**Response: **Hospitals were not the focus as we were looking at primary care level facilities. The hospital was included as additional to the sampling frame, and we have reported it here in the spirit of transparency.

  

- ***Comment:*** *Study participants: The timing is a little unclear for UP-retrospective for Bihar, was that also true for UP? Was there any bias based on availability of the Bihar mentors for interviewing?*

**Response: **It was not retrospective for UP as the project was ongoing. Timing has been shown in the Extended File 1.

  

- ***Comment*** *: It is interesting that no difference was seen between high and low performing sites. I was hoping that the authors would discuss.*

**Response:** Findings: Our objective was not to conduct a comparative study. The reasons for choosing high and low performing facilities have been elaborated further in the manuscript. The differences that emerged have been summarized in the figures in Extended File 2 and explained. However, for the line of inquiry relevant for this paper (making change happen), no differences emerged in how the nurse mentors were able to cope and overcome the barriers they faced. 

  

- **Comment:** One challenge in this model for improvement is when a mentor sees a mistake not being corrected-what were the ethical protections in place of and when the mentor could/would intervene?

**Response:** We appreciate the question and recognise your point. This was indeed an ethical conundrum that some mentors faced; and while we have not reported the specifics in the manuscript, we found that mentors were able to work with the facility staff to correct the smaller mistakes. However, in the few instances where mentors noticed major mistakes that required changes to organisational processes or personnel, they were able to use the office of the TSU to initiate amends – with varying degrees of success.

  

- ***Comment:*** *When the article notes that the mentors were “empowered to go beyond “mere” (interesting choice of words as you note that empathy is important-consider reframing), to take action”-what was that-was it advocating or accessing resources?*

**Response**: Thank you. We agree with you that this framing undermines our argument. We have dropped the word ‘mere.’ Doing so indeed better communicates the finding.

  

- ***Comment:*** *Was the impact on skills as well as the “assertiveness, confidence and self-efficacy” modelled by the mentors? Particularly relevant to the quote from UP and PPH. And the example on MagSulfate.*

**Response**: If we understand your question right, yes, the mentors modelled the skills as well as “assertiveness, confidence and self-efficacy”. The modelling of the “assertiveness, confidence and self-efficacy” was however not an explicit focus of the program, rather it was something that the mentors brought to the program. The importance of these behaviours was recognised by the TSU managers and was incorporated into the support and training package for the mentors.

  

- ***Comment:*** *Did the self-efficacy improvement work in all settings or only those where MOIC or more senior nurses were (or became) welcoming?*

**Response:** We appreciate this question, and we have had much discussion between the co-authors this question. The self-efficacy improvements clearly worked better where the MOIC and the more senior nurses were inherently open and welcoming. But it worked everywhere (all study sites), irrespective of the MOIC and senior nurses, to some extent. In our assessment the latter can to some extent be accounted for by the fact that the broader program (the TSU), was embedded within and was backed by the highest office in the state public health services.

  

- ***Comment*** *: While the results are impressive, it would be important to understand what persisted after the mentorship ended, as other studies have found decrease in impact and some of the quotes discuss the “value of being present” for the mentors (page 7).*

**Response:** We have reflected further on the question of sustainability, and indeed, our findings around the “value of being present” do indicate that not being present would inevitably compromise sustainability.

 While relating what is currently the situation and what continues and persists, is beyond the scope of the current paper, we (co-authors from UP and Bihar), see that the program is learning, including from these findings, and evolving. For instance, the health services in Bihar are in the process of creating an internal mentoring approach for nurses across the services; in UP no clear picture has emerged yet. BMGF India are supporting this evolution.

  

- ***Comment:*** *There is an interesting tension about the potential value of mentors as “outsiders” (page 8) versus the perceptions in earlier sections. It is also important to note that while they may have been outsiders, they were still within the government-endorsed system (TSU) which I suspect may have helped with their acceptance.*

**Response: **We agree, and we thank you for the suggestion to engage with and discuss this tension further. Indeed, had the mentors not been endorsed by the government, they would not have been accepted. We have now explicitly engaged with this tension it in the manuscript, including when discussing the sustainability of the initiative.

  

- ***Comment:*** *The findings are not surprising given the coaching literature and it would be of great value to understand how the programs chose, trained, and mentored the mentors-how was supportive supervision noted in page 6 given earlier, how was the mentor performance assessed over time? The section on how the mentors were trained and supportive is critical and was a little surprised to see at the end of the results. As a reader, I kept looking for that and would have preferred to start with that and a bit more detail in the methods section. Details which are important for replication.*

**Response** *: *Thank you. We see your point. We have elaborated upon this in the Program Design Section and have also referred the reader to the Extended File 1 for further details.

- ***Comment*** *: The details on the “structured” curriculum would be important (perhaps a supplemental file) and example of how they adapted to meet challenges would provide deeper insight into the successes described.*

**Response:** We appreciate your interest in seeing the details of the curriculum. While we have highlighted the key aspects of the two interventions in the Extended File 1, we are not able to share the full curriculum here – hope you understand. If however you would like to access the curriculum, you could write to CARE India, Patna, Bihar, India, or to India Health Action Trust, Lucknow, India. We have indicated this possibility in the Extended File 1. 

- ***Comment*** *: The time and resources required were substantial-is there any costing data to again inform sustainability and replication?*

**Response** *: *Indeed. Time and resources required were substantial given the scale of the intervention. We agree that these details are relevant to any discussion on sustainability, but we do not have access to reliable data.

- ***Comment*** *: I was interested in the concept of “doffing ones hat” and how that persists as a sustainable strategy versus also working with coaching of the MOIC and others to create organizational change over time. Over time for example, did the nurses learn by example and start advocating for change or did that remain in the domain of the mentors?*

**Response: **We see your point, and indeed while it might be a strategy to get one’s way in, it involves a risk of legitimating certain ways of operating and relating which may not be conducive to sustainable organisational. Guided by your suggestions, we have discussed this further in this version of the manuscript.

- ***Comment:*** *The number of interviews is large, but facilities limited, so would be a bit cautious about the “able to win over most skeptics" - that should be caveated by who was interviewed. I also did not hear much from the MOOICs and higher up entrenched individuals about the “unfreezing” and more details would be of great value.*

**Response: ****The number of facilities where the inquiry was conducted are indeed limited, but they are by no means few, and they are as explained, typical of what a public health facility in this part of India would be like.

 We do however agree that only some of the top ranked individuals in the state public health hierarchy were interviewed. The nature of the public service in India is such that authority is concentrated in certain key offices; these officers were the drivers of the initiative and led the TSUs – and they were not the skeptics. The skeptics we refer to, were the middle level officials at the district, sub-district (block) and to some extent health facility levels – and these are typically those who we call ‘Others’ (19 persons) and some from amongst the ‘Program Leaders’ (12 persons).

 Unfreezing is an inference derived primarily from what we observed and what the NMs and TSU staff told us (as having experienced it). It is not something that could be illustrated through quotes from interviews with the aforementioned skeptics. Also, by the time the interviews were done, the program was well advanced, and the skeptics had changed their positions, and there was little point in asking them to discuss their initial positions. 

- ***Comment*** **: **I was also looking for a bit more on the limitations of the approach and data. There is good discussion about the researchers and their experiences, the sustainability unknowns (see above).

**Response: ****We have revised the limitations section further – we explicitly note that our approach (discreet, time limited visits and the reliance on interviews) was itself in some ways a limitation of the study.

- ***Comment:*** *The final conclusions I think are valid, but incomplete and need to acknowledge the resources and time and the knowns about how to sustain both individual as well as the emerging organizational change.*

**Response:** Thank you. While we recognise your point and agree, given the focus of the paper, and given our findings, we think we are not able to conclude about the sustainability of individual and organisational level changes in any further detail. We think that we can only discuss and reflect upon issues of sustainability, and we have tried to do this in the sections that lead up to the conclusions.
